# Effects of inclisiran therapy on metabolomic and lipoproteomic profiles in dyslipidemic patients

**DOI:** 10.3389/fphar.2026.1824522

**Published:** 2026-06-08

**Authors:** Jacopo Patrussi, Alessia Vignoli, Leonardo Tenori, Andrea Sorrentino, Martina Berteotti, Elena Lotti, Felice Crudele, Giulia Ciarrocca Taranta, Angela Antonietta Rogolino, Giulia Barbieri, Anna Maria Gori, Rossella Marcucci

**Affiliations:** 1 Department of Chemistry “Ugo Schiff”, University of Florence, Florence, Italy; 2 Magnetic Resonance Center (CERM), University of Florence, Florence, Italy; 3 Department of Experimental and Clinical Medicine, University of Florence, Florence, Italy; 4 Azienda Ospedaliero-Universitaria Careggi, Florence, Italy

**Keywords:** dyslipidemia, lipoproteomics, metabolomics, NMR spectroscopy, PCSK9 inhibitors

## Abstract

**Background:**

Inclisiran is a subcutaneously administered synthetic small interfering RNA directed against proprotein convertase subtilisin–kexin type 9, leading to sustained low-density lipoprotein cholesterol (LDL-C) reduction with a twice-yearly dosing regimen. While its lipid-lowering efficacy is well established, its broader metabolic effects remain incompletely characterized. This study aimed to comprehensively evaluate serum metabolomic and lipoproteomic changes in patients with dyslipidemia before and after inclisiran treatment using an integrated nuclear magnetic resonance (NMR)-based approach.

**Methods:**

This observational single-center study included 69 patients with dyslipidemia treated with inclisiran in routine clinical practice. Fasting serum samples were collected at baseline and 3 months after treatment initiation. A total of 30 metabolites and 112 lipoprotein-related parameters were quantified using NMR spectroscopy. Paired comparisons between time points were performed using the Wilcoxon signed-rank test with false discovery rate correction. Clustering based on lipoprotein changes was conducted using the KODAMA algorithm to identify distinct response patterns.

**Results:**

Inclisiran treatment was associated with extensive and coherent reductions in lipoprotein-related parameters. Seventy-four lipoprotein variables showed significant changes. Reductions were observed across LDL, IDL, and VLDL subclasses, while some HDL-related parameters were increased. In contrast, the global metabolomic profile remained largely unchanged; trimethylamine-N-oxide (TMAO) was the only metabolite significantly increased at follow-up. KODAMA clustering identified three patient groups with heterogeneous responses. Two groups demonstrated marked lipoprotein reductions, particularly those with higher baseline LDL-related parameters, whereas one group exhibited minimal changes. Baseline TMAO levels were lower in the group with the most pronounced lipid response and increased at follow-up, potentially reflecting changes in concomitant lipid-lowering therapy rather than a direct effect of inclisiran.

**Conclusion:**

Inclisiran induces a broad and consistent improvement in lipoprotein profiles, extending beyond LDL-C reduction, with minimal impact on the overall metabolome. These findings support the highly specific lipid-lowering action of inclisiran and highlight inter-individual variability in treatment response, especially in concomitant lipid-lowering therapies.

## 1 Introduction

Low-density lipoprotein cholesterol (LDL-C) has been consistently shown to be causally associated with the risk of atherosclerotic cardiovascular disease. This means that lowering circulating LDL-C levels over the long-term is critical for the prevention of atherosclerotic events (Mach et al., 2020). Daily administration of statins has long been the first-line therapy for the long-term management of LDL-C levels. However, statin treatment does not always result in the achievement of the recommended LDL-C concentrations, especially in very high-risk patients; moreover, poor adherence and statin intolerance may further adversely affect cardiovascular outcomes. Inclisiran is a more recent lipid-lowering medication that has been shown to be effective in lowering LDL-C levels during prolonged follow-up periods with twice-yearly dosing regimen (Ray et al., 2023; Wright et al., 2024). Inclisiran is a subcutaneously administered synthetic small interfering RNA (siRNA) directed against proprotein convertase subtilisin–kexin type 9 (PCSK9) (Khvorova, 2017). The latter is a serine protease that binds the LDL receptor in hepatocytes, promoting receptor degradation; as a result, this mechanism leads to an increase in LDL-C levels in the bloodstream. The siRNA molecules are conjugated to triantennary N-acetylgalactosamine (GalNAc), which specifically binds to asialoglycoprotein receptors, which are highly expressed on the surface of hepatocytes responsible for cholesterol uptake, leading to high specificity targeting. At molecular level, inclisiran therapeutic effect is based on the RNA interference mechanism: siRNA induces the cleavage of the messenger RNA (mRNA) encoding PCSK9 through the RNA-induced silencing complex, resulting in reduced levels of PCSK9 protein. Thus, inclisiran carry out a long lasting and effective reduction of serum LDL-C levels (Fitzgerald et al., 2017; Khvorova, 2017).

Evaluating patient’s drug response is crucial for assessing personalized drug prescription, as well as identifying possible toxicity or lack of efficacy. In this context, metabolomics not only enable the molecular characterization of patients within the dynamic context of a disease process but also can provide a comprehensive framework for capturing the physiological states of patients during therapy, thereby informing personalized therapeutic strategies (Wishart, 2016; Vignoli et al., 2020; 2022; Gonzalez-Covarrubias et al., 2022). In this study, we used ^1^H-NMR spectroscopy to obtain both metabolomic and lipoproteomic profiles from serum samples of 69 patients before and after inclisiran treatment, with the aim of characterizing treatment-associated changes in circulating metabolites and lipoprotein subclasses.

## 2 Materials and methods

### 2.1 Ethical statements

The study was conducted in accordance with the principles of the Declaration of Helsinki and Good Clinical Practice guidelines. The study protocol (“Characterization of the pleiotropic effects of PCSK9 inhibitors”), including all procedures involving human participants and biological sample collection, was reviewed and approved by the Ethics Committee of the Regione Toscana–Area Vasta Centro (CEAVC) (approval ID: 2024-079; approval date: 23 July 2024).

All participants provided written informed consent prior to enrollment. Consent included authorization for the collection, storage, and analysis of biological samples, as well as for the anonymized processing of clinical and laboratory data for research purposes. Patient data were handled in compliance with applicable national and European data protection regulations, and all analyses were performed on anonymized datasets.

### 2.2 Study cohort and sample collection

This observational, single-center study included 69 adult patients, both men (41) and women (28), referred to the Atherothrombotic Diseases Unit (SOD Malattie Aterotrombotiche) of Azienda Ospedaliero-Universitaria Careggi, Florence, Italy, for the management of dyslipidemia. Patients were treated in the context of primary or secondary cardiovascular prevention and included individuals with heterozygous familial hypercholesterolemia (HeFH), non-familial hypercholesterolemia, or mixed dyslipidemia.

All patients started therapy with inclisiran as part of routine clinical practice, according to the reimbursement criteria established by the Italian Medicines Agency, which also defined the inclusion criteria for the present investigation. Briefly, eligible patients were aged ≤80 years and fulfilled at least one of the following conditions: (i) primary prevention patients with HeFH and LDL-C ≥130 mg/dL despite at least 6 months of high-potency statin therapy at the maximum tolerated dose combined with ezetimibe, or documented intolerance to statins and/or ezetimibe; (ii) secondary prevention patients with HeFH, non-familial hypercholesterolemia, or mixed dyslipidemia and LDL-C ≥70 mg/dL despite optimized lipid-lowering therapy, or patients with recent myocardial infarction (within the previous 12 months), multiple cardiovascular events, or documented intolerance to statins and/or ezetimibe.

The decision to prescribe inclisiran was made independently of study participation and was based exclusively on clinical judgment within the department’s standard care pathways. Inclisiran was administered by healthcare personnel in an outpatient setting following the approved dosing schedule.

Serum samples were collected at two predefined time points. Baseline blood sampling (t0) was performed on the day of the first inclisiran administration, prior to drug injection, in order to capture the biological profile at treatment initiation. Follow-up sampling (t1) was conducted 3 months later, on the day of the second inclisiran administration. Both sampling time points coincided with scheduled clinical visits, ensuring standardized timing and minimizing additional patient burden. Venous blood samples were collected under fasting conditions as part of routine laboratory assessments. Serum was separated using standard clinical laboratory procedures and stored under controlled conditions until subsequent analyses. All samples were anonymized through the assignment of a unique alphanumeric study code to ensure patient confidentiality.

### 2.3 NMR analysis

Serum samples were thawed at room temperature and vortex-mixed prior to preparation: 350 µL of sodium phosphate buffer (75 mM Na_2_HPO_4_ × 7H_2_O; 20% (v/v) ^2^H_2_O, 4.6 mM 3-(Trimethylsilyl) propionate- 2,2,3,3-d4; 6.1 mM NaN_3_; pH 7.4) was added to 350 µL of each sample, followed by a vortex homogenization and 600 µL of each mixture was transferred into a 5 mm NMR tube.

A Bruker 600 MHz spectrometer (Bruker Biospin) operating at 600.13 MHz proton Larmor frequency and equipped with a 5 mm PXTI ^1^H-^13^C-^15^N and ^2^H-decoupling probe including a z-axis gradient coil, an automatic tuning-matching (ATM) unit and an automatic refrigerated (6 °C) sample changer (SampleJet, Bruker Biospin) was used to acquire ^1^H-NMR spectra for all samples. A BTO 2000 thermocouple was used to stabilize the sample temperature at the level of approximately 0.1 K. Before NMR acquisition, each sample was maintained inside the NMR probe head for at least 300 s to allow temperature equilibration at 310 K. For each serum sample, a standard nuclear Overhauser effect spectroscopy (NOESY) pulse sequence was applied using 32 scans, 98,304 data points, a spectral width of 18,028 Hz, an acquisition time of 2.7 s, a relaxation delay of 4 s and a mixing time of 0.01 s to detect the NMR signals of both high and low molecular weight molecules (Ghini et al., 2023). Before Fourier transformation, the free induction decays were multiplied by an exponential function corresponding to a 0.3 Hz line-broadening factor. Transformed spectra were automatically corrected for phase and baseline distortions and calibrated to the anomeric glucose doublet at δ 5.24 ppm.

Metabolites and lipoprotein-related parameters were quantified from the same ^1^H-NMR spectra, using two platforms extracting complementary information from the same spectral dataset. Through the Bruker IVDr Quantification platform for Plasma/Serum (B.I.Quant-PSTM™, version 2.1.0) a panel of 20 metabolites was identified and quantified. In addition, the signals of glycoproteins GlycA at δ 2.04 and GlycB at δ 2.08 ppm, together with other eight metabolites, were quantified by integration using an in-house developed R script. Furthermore, the Bruker IVDr Lipoprotein Subclass Analysis platform™ (version 1.1.0) was used to quantify 112 lipoprotein-related parameters. These parameters include the concentration of triglycerides, cholesterol, free cholesterol, phospholipids, Apo-A1, Apo-A2 and Apo-B100 of the main fraction and subfraction of HDL, LDL, IDL and VLDL classes (Jiménez et al., 2018).

### 2.4 Statistical analysis

All data analyses were conducted in the “R” statistical environment. Only patients with samples collected at both time points were considered for the following analysis. Wilcoxon signed-rank test was applied to assess paired differences in metabolites and lipoproteins between baseline (t0) and 3 months after treatment (t1).

The arithmetic difference between t1 and t0 was calculated for each lipoprotein-related parameter and this matrix was used to identify potential clusters present in the dataset using the KODAMA algorithm. This method performs feature extraction from high-dimensional data and performs cross-validation of the results to maximize the accuracy of the prediction (Cacciatore et al., 2014; Zinga et al., 2022). Three different groups of patients (1, 2 and 3) were identified with this approach. The Wilcoxon signed-rank test was used to compare lipoprotein and metabolite levels between the two time points in each KODAMA group. Moreover, to investigate possible associations between baseline metabolite levels and the KODAMA groups, the Wilcoxon rank sum test was used. Lastly, non-parametric tests were used to assess the presence of significant differences in frequency distributions in clinical variables among KODAMA groups. The chi-square test was employed for variables with expected frequencies higher than 5, whereas Fisher’s exact test for frequency lower than 5. All *p*-values were adjusted for multiple testing using the false discovery rate (FDR) procedure with Benjamini–Hochberg correction at α = 0.05.

## 3 Results

### 3.1 Study cohort characteristics

The analyses were conducted on 69 patients with hypercholesterolemia. Baseline demographic, clinical and pharmacological information of the entire study cohort are summarized in Table 1. The table includes information on cardiovascular history, lipid-related disorders and the different lipid-lowering treatments received prior to inclisiran therapy.

**TABLE 1:** Demographic and clinical characteristics of the baseline study cohort (n = 69).

Patient information	Study cohort (n = 69)
Demographic characteristics	
Age, median (IQR)	60 (54–70)
Sex (female), n (%)	28 (40.6)
Current smokers, n (%)	16 (24.6)
Ex-smokers, n (%)	24 (36.9)
Clinical history	
Hypertension, n (%)	35 (53.8)
Diabetes, n (%)	11 (16.9)
Hyperuricemia, n (%)	3 (4.6)
Family history of CAD, n (%)	30 (46.2)
Family history of cerebrovascular disease, n (%)	4 (6.2)
Stable CAD, n (%)	16 (24.6)
Unstable angina, n (%)	5 (7.7)
Myocardial infarction, n (%)	28 (43.1)
PCI, n (%)	31 (47.7)
Bypass, n (%)	4 (6.2)
Stroke/TIA, n (%)	1 (1.5)
Carotid artery disease, n (%)	10 (15.4)
Peripheral arterial disease, n (%)	7 (10.8)
Peripheral revascularization, n (%)	2 (3.1)
TEA, n (%)	2 (3.1)
Heart failure, n (%)	3 (4.6)
Renal function, n (%)	3 (4.6)
Hepatic function, n (%)	1 (1.5)
HeFH, n (%)	26 (40)
Non-familial hypercholesterolemia, n (%)	34 (52.3)
Mixed dyslipidemia, n (%)	16 (24.6)
LDL-C above target, n (%)	49 (75.4)
Statin intolerance, n (%)	31 (47.7)
Poor adherence to PCSK9 inhibitors, n (%)	2 (3.1)
Lipid-lowering medications	
Atorvastatin, n (%)	12 (18.5)
Rosuvastatin, n (%)	32 (49.2)
Simvastatin, n (%)	3 (4.6)
Ezetimibe, n (%)	51 (78.5)
PUFA, n (%)	2 (3.1)
Bempedoic acid, n (%)	13 (20)
Other PCSK9 inhibitors, n (%)	5 (7.7)

IQR, interquartile range; CAD, coronary artery disease; PCI, percutaneous coronary intervention; TIA, transient ischemic attack; TEA, thromboendarterectomy; HeFH, heterozygous familial hypercholesterolemia; PUFA, polyunsaturated fatty acids.

### 3.2 Univariate analysis of metabolites and lipoproteins after treatment

**FIGURE 1:**
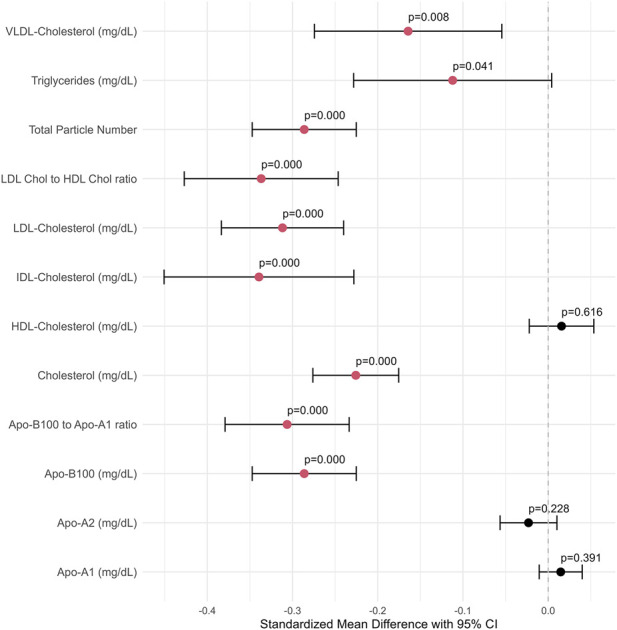
Lipoprotein main parameters standardized mean differences between t1 and t0 samples. Negative values indicate a decrease at t1. Red dots indicate statistically significant parameters (*p*-value FDR adjusted <0.05).

Changes in 30 metabolites and 112 lipoprotein parameters were evaluated using the Wilcoxon signed-rank test, comparing t0 and t1 levels. Among metabolites, only trimethylamine-N-oxide (TMAO) was found to be significantly different between the two time-points, with increased levels at t1 (Supplementary Table S1). In contrast, extensive alterations in the lipoprotein profiles were detected with 74 out of 112 lipoprotein parameters statistically different (Supplementary Table S1). Among the main lipoprotein parameters: Total Particle Number, LDL-C, Cholesterol, Apo-B100, Triglycerides, LDL-C to HDL cholesterol (HDL-C) ratio and Apo-B100 to Apo-A1 ratio reported significant reductions (Figure 1). Regarding subfractions, four parameters were increased at t1 all related to HDL-C, specifically Apo-A1 in HDL-1 and HDL-2, phospholipids in HDL-1, and free cholesterol in HDL-3 (Supplementary Figure S1; Supplementary Table S1). Regarding lipoprotein subfractions all LDL-related parameters were significantly decreased (Supplementary Figure S2; Supplementary Table S1), together with most IDL (Supplementary Figure S3; Supplementary Table S1) and VLDL fractions (Supplementary Figure S4; Supplementary Table S1).

### 3.3 KODAMA clustering and metabolic changes

**FIGURE 2:**
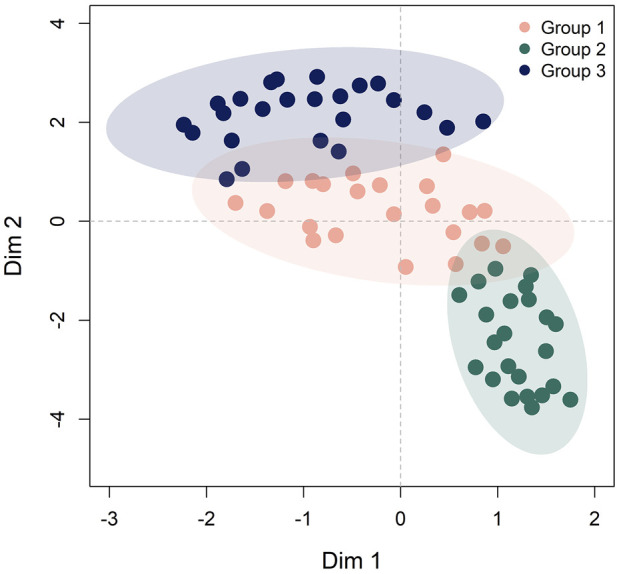
Visual representation of KODAMA clustering in the matrix of lipoprotein differences, identifying three groups of patients: group 1 (light pink), group 2 (green), group 3 (blue).

KODAMA algorithm, applied to the matrix of lipoprotein differences, identified three well defined clusters of patients (Figure 2). Notably, the use of intra-individual differences (arithmetic difference between t1 and t0) helps controls for inter-individual variability, thereby limiting the effect of some potential confounding factors. The three groups, named 1, 2 and 3, were constituted by 22, 23 and 24 patients, respectively.

**FIGURE 3:**
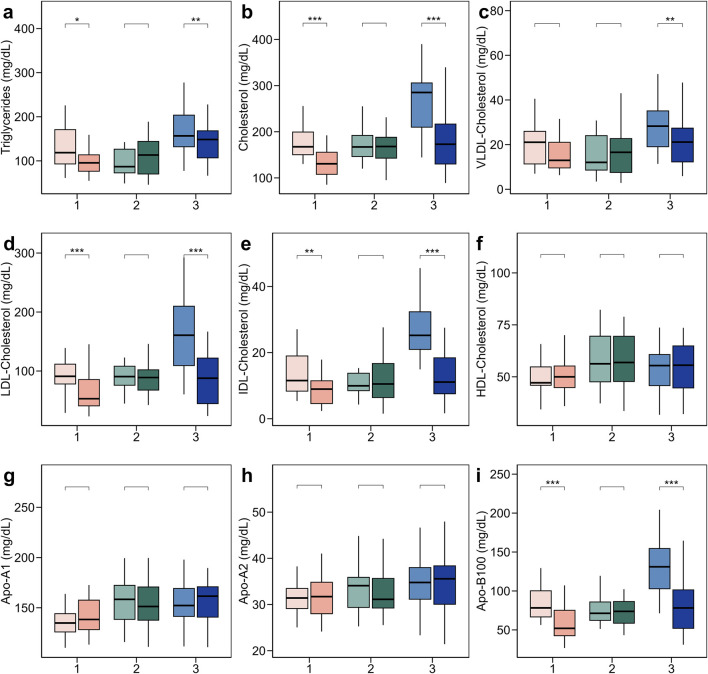
Levels of the main lipoprotein-related parameters in the three KODAMA groups of patients (group 1: pink, group 2: green, group 3: blue) at the two time points (t0: light color; t1: dark color): **(a)** triglycerides, **(b)** cholesterol, **(c)** VLDL cholesterol, **(d)** LDL cholesterol, **(e)** IDL cholesterol, **(f)** HDL cholesterol, **(g)** Apo A1, **(h)** Apo A2, **(i)** Apo B100. *p*-values obtained using Wilcoxon signed-rank test and adjusted for FDR are reported. ***p < 0.001; **p < 0.01; *p < 0.05.

Examination of the trends in each group, particularly for LDL-related parameters, suggested that patients in the third group started from higher baseline levels of these fractions and showed a more pronounced reduction after treatment. Group 1 displayed a similar tendency, despite having lower baseline levels. In contrast to groups 1 and 3, group 2 showed little to no differences after the treatment (Figure 3).

**FIGURE 4:**
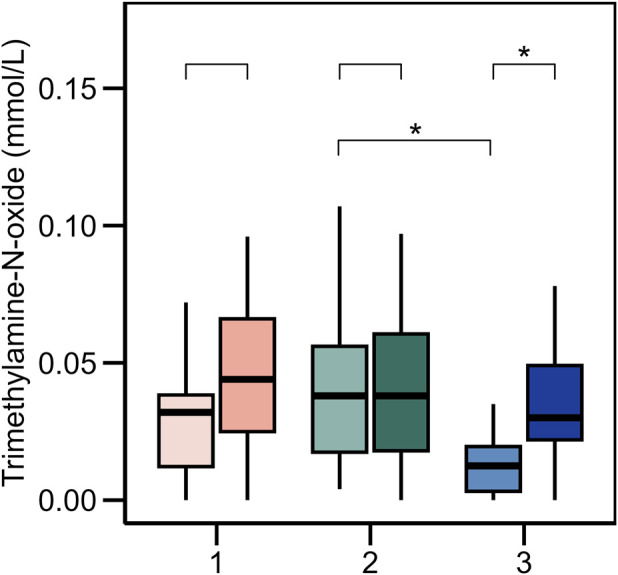
Levels of Trimethylamine-N-oxide in the three KODAMA groups (group 1: pink, group 2: green, group 3: blue) at the two time points (t0: light color; t1: dark color). *p*-values obtained using Wilcoxon test and adjusted for FDR are reported. ***p < 0.001; **p < 0.01; *p < 0.05.

To study the trends of metabolites and lipoproteins levels after the treatment, in each KODAMA group, the Wilcoxon signed-rank test was applied (Supplementary Table S2). Groups 1 and 3 showed marked alterations in several lipoprotein-related parameters between baseline and post-treatment. In group 3, 68 lipoprotein-related parameters differed significantly between the two timepoints. Among these, only Apo-A1 in HDL-1 and HDL-2 increased at t1. Conversely, seven main parameters (Total Particle Number, Triglycerides, Cholesterol, LDL-C, Apo-B100, LDL-C to HDL-C ratio and Apo-B100 to Apo-A1 ratio), together with 35 LDL-, 18 VLDL-, and all 6 IDL-related parameters, showed significant decreases. Similarly, in group 1, 53 lipoprotein-related parameters were significantly reduced at t1, including the same seven main parameters, most LDL- (25) and VLDL- (15) parameters, and all IDL-related parameters (6). In contrast, group 2 did not show any significant differences between the two timepoints. Among the metabolites, trimethylamine-N-oxide was the only compound showing a significant change, with increased levels at t1 in group 3. In addition, baseline trimethylamine N-oxide levels were significantly lower in group 3 than in group 2 (Figure 4). No other metabolites showed significant variations across the three groups.

The demographic characteristics and clinical history of patients clustered in the three KODAMA groups are reported in Table 2. These data show that group 3 includes a slightly higher proportion of patients with heterozygous familial hypercholesterolemia and lower proportion of patients with non-familial hypercholesterolemia, whereas groups 1 and 2 include a higher proportions of patients with recent atherosclerotic vascular disease. At baseline, patients in the three KODAMA groups were receiving a variety of cholesterol-lowering therapies, including atorvastatin, rosuvastatin, simvastatin, ezetimibe, polyunsaturated fatty acids (PUFA) supplements, bempedoic acid, and other PCSK9 inhibitors. The proportion of patients on these therapies varied across groups, with ezetimibe and statins being the most used. Across the follow-up period, the distribution of most treatments remained largely stable, with some minor changes, such as an increase of ezetimibe use in group 3 (from 72.3% to 86.4%). Non-parametric test revealed no significant differences in the distribution in clinical variables among KODAMA groups. Overall, these data provide a comprehensive overview of concomitant lipid-lowering treatments across groups and timepoints.

**TABLE 2:** Demographic characteristics and clinical history of patients in the three KODAMA groups.

Kodama	Group 1	Group 2	Group 3	FDR adj. *p*-value
Demographic characteristics				
Sex (female), n (%)	4 (18.2)	10 (43.5)	14 (58.3)	0.216*
Current smokers, n (%)	4 (17.4)	3 (13.6)	10 (45.5)	0.216*
Ex-smokers, n (%)	12 (52.2)	8 (36.4)	5 (22.7)	0.391
Clinical history				
High risk, n (%)	3 (15)	6 (28.6)	11 (55)	0.216*
Very high risk, n (%)	17 (85)	15 (71.4)	9 (45)	0.216*
Hypertension, n (%)	13 (61.9)	11 (50)	11 (50)	0.928
Diabetes, n (%)	6 (28.6)	2 (9.1)	3 (13.6)	0.574
Family history of CAD, n (%)	12 (57.1)	10 (47.6)	8 (36.4)	0.710
Family history of cerebrovascular disease, n (%)	2 (9.5)	1 (4.7)	1 (4.5)	0.973
Stable CAD, n (%)	6 (28.6)	8 (36.4)	2 (9.1)	0.368
Unstable angina, n (%)	1 (4.7)	2 (9.1)	2 (9.1)	1.00
Myocardial infarction, n (%)	13 (61.9)	9 (40.9)	6 (27.3)	0.332
PCI, n (%)	14 (66.7)	11 (50)	6 (27.3)	0.216*
Bypass, n (%)	2 (9.5)	2 (9.1)	0 (0)	0.735
Stroke/TIA, n (%)	1 (4.7)	0 (0)	0 (0)	0.614
Carotid artery disease, n (%)	3 (14.3)	3 (13.6)	4 (18.2)	1.00
Peripheral arterial disease, n (%)	1 (4.7)	3 (13.6)	3 (13.6)	0.928
Peripheral revascularization, n (%)	0 (0)	0 (0)	2 (9.1)	0.614
TEA, n (%)	1 (4.7)	1 (4.5)	0 (0)	1.00
Heart failure, n (%)	1 (4.7)	2 (9.1)	0 (0)	0.813
HeFH, n (%)	5 (23.8)	8 (36.4)	13 (59.1)	0.305
Non-familial hypercholesterolemia, n (%)	15 (71.4)	12 (54.5)	7 (31.8)	0.216*
Mixed dyslipidemia, n (%)	4 (19)	4 (18.2)	8 (36.4)	0.612
LDL-C above target, n (%)	17 (81)	16 (72.7)	16 (72.7)	0.946
Statin intolerance, n (%)	7 (33.3)	10 (45.5)	14 (63.6)	0.391
Poor adherence to PCSK9 inhibitors, n (%)	1 (4.7)	1 (4.5)	0 (0)	0.946
Lipid-lowering medications				
Atorvastatin, n (%) baseline	4 (19)	5 (22.7)	3 (13.6)	0.946
Atorvastatin, n (%) follow up	4 (19)	4 (18.2)	3 (13.6)	0.973
Rosuvastatin, n (%) baseline	14 (66.7)	10 (45.4)	8 (36.4)	0.391
Rosuvastatin, n (%) follow up	13 (61.9)	10 (45.4)	8 (36.4)	0.536
Simvastatin, n (%) baseline	1 (4.8)	2 (9.1)	0 (0)	0.717
Simvastatin, n (%) follow up	0 (0)	1 (4.5)	0 (0)	0.928
Ezetimibe, n (%) baseline	19 (90.5)	16 (72.7)	16 (72.3)	0.593
Ezetimibe, n (%) follow up	21 (100)	17 (77.3)	19 (86.4)	0.352
PUFA, n (%) baseline	0 (0)	1 (4.5)	1 (4.5)	1.00
PUFA, n (%) follow up	0 (0)	1 (4.5)	0 (0)	1.00
Bempedoic acid, n (%) baseline	6 (28.6)	4 (18.2)	3 (13.6)	0.735
Bempedoic acid, n (%) follow up	7 (33.3)	10 (45.4)	4 (18.2)	0.415

FDR, false discovery rate; CAD, coronary artery disease; PCI, percutaneous coronary intervention; TIA, transient ischemic attack; TEA, thromboendarterectomy; HeFH, heterozygous familial hypercholesterolemia; PUFA, polyunsaturated fatty acids.

*P*-values marked with * indicates variables significant before FDR correction.

## 4 Discussion

In this study, an extensive metabolomic and lipoproteomic analysis was performed to characterize the serum metabolic changes in patients with dyslipidemia after treatment with inclisiran. The focal finding is a coherent and widespread alteration of lipoprotein parameters related to LDL, VLDL and IDL, with little increase of some HDL-related parameters after treatment. Whereas, serum metabolome appears to be only minimally affected by the treatment, with trimethylamine-N-oxide occurring as the only metabolite significantly different after inclisiran administration.

Univariate analysis revealed a significant reduction in a large number of lipoprotein-related parameters following inclisiran treatment. These changes affected not only conventional lipid markers, including total cholesterol, LDL-C, HDL-C, Apo-B, and triglycerides, but also several lipoprotein subfractions. At the same time, some HDL-related parameters appear increased after treatment. These findings outline the known mechanism of inclisiran, which selectively increases the clearance of LDL particles from blood circulation degrading mRNA encoding PCSK9. The simultaneous reduction of LDL, IDL and VLDL fractions sustains the evidence that this drug not only acts his action on LDL but also in others ApoB-containing lipoproteins (Warden and Duell, 2021).

Despite the large modification of the lipoprotein profile, metabolites appear to be largely unaffected by the treatment, with trimethylamine-N-oxide being the only one significantly increased after treatment in a subgroup of patients. This suggests that inclisiran does not affect metabolomic profiles outside of lipoprotein-related parameters, reflecting the specific action of this drug against PCSK9, due to siRNAs intrinsic specificity (Khvorova, 2017).

The KODAMA algorithm enables the stratification of patients treated with inclisiran in three groups based on lipoprotein changes, pointing out an inter-individual variability in treatment response. Patients of group 3 showed higher baseline levels of LDL-, IDL-, VLDL-related parameters, triglycerides and total cholesterol, with a marked reduction of these variables after treatment. Patients of group 1 exhibited a similar pattern, even though with lower basal levels. On the contrary, patients of group 2 revealed minimal changes in lipoprotein profiles after treatment, indicating a limited response to the treatment. Accordingly, as reported in Table 2, group 3 includes a higher proportion of patients with HeFH, which have notoriously higher cholesterol levels, but a lower proportion of patients with non-familial hypercholesterolemia. On the other hand, groups 1 and 2 include a higher proportion of patients with recent atherosclerotic vascular diseases, in the subacute phase of the disease.

Group 3 patients showed lower basal levels of trimethylamine-N-oxide that subsequently increased at follow-up after 3 months of inclisiran therapy. Notably, in this group the final TMAO levels were not significantly different from the other two groups and we documented an increase as the basal TMAO levels were significantly lower. There is robust evidence of the association between high levels of TMAO and multiple diseases, including cardiovascular diseases (Li et al., 2022; Xiong et al., 2022). In the pathogenesis of these diseases, the inflammatory response represents a key mechanism. Given the ability of TMAO to regulate inflammation, together with evidence supporting the involvement of the intestinal microbiota through the gut–brain axis, TMAO may play a role in the modulation of immune responses in these conditions. (Saaoud et al., 2023). A significant amount of trimethylamine is synthesized by the gut microbiota and absorbed from the intestinal lumen into the portal circulation by passive diffusion across enterocytes, and is subsequently oxidized to TMAO in the liver (Kałużna-Czaplińska and Gątarek, 2021; Liu et al., 2025). In our population, baseline TMAO levels differed among groups. In particular, significantly lower levels were found in group 3 as compared to group 2. These findings are likely related to the fact that group 3 mainly includes patients with heterozygous familial hypercholesterolemia, without a recent cardiovascular event. In contrast, groups 1 and 2 mainly include patients with a recent cardiovascular or cerebrovascular event, suggesting that the recent atherothrombotic process is associated with an inflammatory process with higher TMAO levels.

At the sampling point three months after the first administration of inclisiran, TMAO levels remained virtually unchanged in group 2; they show a slight (non-significant) increase in group 1 and increase significantly in group 3. Since inclisiran administration was the same (in terms of timing and dosage) in all three groups, this change was clearly not attributable to inclisiran administration. We therefore looked for other possible clinical variables that could be associated with this change. The only associated variable was identified in the percentage of patients who started taking ezetimibe after the first blood sample was collected. In fact, in both group 1 and group 3, a higher percentage of patients started taking ezetimibe, which was more significant in group 3 (from 72.7% to 86.4%) than in group 1 (from 90.5% to 100%). Since the gut microbiota is one of the main determinants of circulating TMAO levels, we hypothesized that taking ezetimibe, which interferes with intestinal cholesterol absorption (Ge et al., 2008), would cause certain changes that could affect TMAO levels. This was confirmed in group 2, where there was no change in TMAO levels and the percentage of ezetimibe intake remained virtually constant (from 72.7% to 77.3%).

To the best of our knowledge, this is the first study applying an NMR-based metabolomic and lipoproteomic approach to investigate the systemic metabolic effects of inclisiran therapy. However, as a preliminary study, several limitations should be acknowledged. In particular, the observational single-center design and the relatively small sample size limit the ability to draw causal inferences regarding the effects of inclisiran on the metabolomic and lipoprotein profiles. Moreover, the absence of a control group or comparator arm (e.g., statin-only therapy or alternative PCSK9 inhibitor treatment) further restricts the interpretability of the findings, which should therefore be considered primarily descriptive. Finally, although patients were receiving different concomitant lipid-lowering therapies, the limited sample size precluded meaningful stratification according to background treatments, which may have contributed to residual heterogeneity in the observed metabolic profiles. However, no significant variations among the three KODAMA groups (Table 2) in terms of clinical and pharmacological characteristics strongly suggest that variations in lipoproteins concentrations were mainly driven by inclisiran administration.

In conclusion, these results do not show any pleiotropic effects of inclisiran beyond its powerful lipid-lowering effect. This finding is consistent with the drug’s action being exclusively targeted at blocking PCSK9 mRNA expression. On the other hand, this metabolomic study highlights the changes of a parameter associated with increased cardiovascular risk, which may be linked to the effects of ezetimibe on the gut microbiota. However, this is a “hypothesis-generating” observation that requires further, specific investigation.

## Data Availability

The data presented in the study are deposited in the Figshare repository, accession number https://doi.org/10.6084/m9.figshare.31899091.
