# Supplementary material for: Effects of inclisiran therapy on metabolomic and lipoproteomic profiles in dyslipidemic patients
Source: Front Pharmacol. 2026 Jun 8;17:1824522. doi: 10.3389/fphar.2026.1824522 (PMC13284123; doi:10.3389/fphar.2026.1824522)
Supplement: Supplementary file 1 [file Supplementaryfile1.docx]

Supplementary Material

# Supplementary Figures and Tables

**Supplementary Table 1.** Univariate analysis of quantified lipoproteins. Trimethylamine-N-oxide is reported in this table as the only significant metabolite. Median values of particle number parameters are expressed in nmol/L, Trimethylamine-N-oxide in mmol/L and all others lipoprotein parameters in mg/dL. The p-values obtained using Wilcoxon signed-rank test are provided also adjusted for FDR are reported. *** p < 0.001; ** p < 0.01; * p < 0.05; ns p ≥ 0.05.

|  | **Median t0** | **Median t1** | ***P*-value** | **FDR adj. *P*-value** |
| --- | --- | --- | --- | --- |
| **Trimethylamine-N-oxide** | 0.027 | 0.035 | *** | * |
| **Main Parameter, TG** | 127.75 | 111.98 | * | * |
| **Main Parameters, Chol** | 192.15 | 156.07 | *** | *** |
| **Main Parameters, LDL-Chol** | 99.58 | 78.37 | *** | *** |
| **Main Parameters, HDL-Chol** | 51.46 | 53.92 | ns | ns |
| **Main Parameters, Apo-A1** | 144.22 | 150.57 | ns | ns |
| **Main Parameters, Apo-A2** | 33.43 | 31.94 | ns | ns |
| **Main Parameters, Apo-B100** | 86.1 | 64.95 | *** | *** |
| **LDL Chol to HDL Chol ratio** | 2.04 | 1.36 | *** | *** |
| **Apo-B100 to Apo-A1 ratio** | 0.59 | 0.43 | *** | *** |
| **Total Particle Number** | 1565.49 | 1180.9 | *** | *** |
| **VLDL Particle Number** | 170.18 | 148.8 | ** | ** |
| **IDL Particle Number** | 125.04 | 88.23 | *** | *** |
| **LDL Particle Number** | 1216.06 | 940.8 | *** | *** |
| **LDL-1 Particle Number** | 182.29 | 142.66 | *** | *** |
| **LDL-2 Particle Number** | 153.05 | 122.66 | *** | *** |
| **LDL-3 Particle Number** | 165.9 | 122.68 | *** | *** |
| **LDL-4 Particle Number** | 208.58 | 134.12 | *** | *** |
| **LDL-5 Particle Number** | 259.86 | 180.38 | *** | *** |
| **LDL-6 Particle Number** | 311.76 | 242.27 | *** | *** |
| **Triglycerides, VLDL** | 85.25 | 75.91 | ns | ns |
| **Triglycerides, IDL** | 10.17 | 7.62 | * | ns |
| **Triglycerides, LDL** | 24.08 | 19.57 | *** | *** |
| **Triglycerides, HDL** | 12.41 | 12.13 | ns | ns |
| **Cholesterol, VLDL** | 20.87 | 15.74 | ** | ** |
| **Cholesterol, IDL** | 15.44 | 10.49 | *** | *** |
| **Free Cholesterol, VLDL** | 9.38 | 8.01 | ** | * |
| **Free Cholesterol, IDL** | 4.29 | 2.73 | *** | *** |
| **Free Cholesterol, LDL** | 32.15 | 27.9 | *** | *** |
| **Free Cholesterol, HDL** | 13.69 | 14.16 | ns | ns |
| **Phospholipids, VLDL** | 21.42 | 18.16 | * | * |
| **Phospholipids, IDL** | 7.91 | 5.12 | *** | *** |
| **Phospholipids, LDL** | 58.33 | 48.16 | *** | *** |
| **Phospholipids, HDL** | 67.37 | 70.47 | * | ns |
| **Apo-A1, HDL** | 140.52 | 148.97 | ns | ns |
| **Apo-A2, HDL** | 33.59 | 32.69 | ns | ns |
| **Apo-B, VLDL** | 9.36 | 8.18 | ** | ** |
| **Apo-B, IDL** | 6.88 | 4.85 | *** | *** |
| **Apo-B, LDL** | 66.88 | 51.74 | *** | *** |
| **Triglycerides, VLDL-1** | 41.01 | 39.01 | ns | ns |
| **Triglycerides, VLDL-2** | 11.07 | 9.07 | ns | ns |
| **Triglycerides, VLDL-3** | 11.45 | 8.99 | * | * |
| **Triglycerides, VLDL-4** | 10.32 | 8.18 | *** | *** |
| **Triglycerides, VLDL-5** | 2.55 | 2.44 | * | * |
| **Cholesterol, VLDL-1** | 6.21 | 5.48 | ns | ns |
| **Cholesterol, VLDL-2** | 2.81 | 1.85 | ** | ** |
| **Cholesterol, VLDL-3** | 3.57 | 2.71 | ** | ** |
| **Cholesterol, VLDL-4** | 5.12 | 3.58 | *** | *** |
| **Cholesterol, VLDL-5** | 0.78 | 0.47 | ** | * |
| **Free Cholesterol, VLDL-1** | 1.82 | 1.75 | ns | ns |
| **Free Cholesterol, VLDL-2** | 1.45 | 1.04 | * | * |
| **Free Cholesterol, VLDL-3** | 1.65 | 1.34 | * | * |
| **Free Cholesterol, VLDL-4** | 2.5 | 1.71 | *** | *** |
| **Free Cholesterol, VLDL-5** | 0.34 | 0.24 | * | ns |
| **Phospholipids, VLDL-1** | 5.99 | 5.99 | ns | ns |
| **Phospholipids, VLDL-2** | 3.04 | 2.37 | ns | ns |
| **Phospholipids, VLDL-3** | 3.82 | 3.02 | * | * |
| **Phospholipids, VLDL-4** | 5.3 | 3.84 | *** | *** |
| **Phospholipids, VLDL-5** | 1.15 | 0.94 | * | ns |
| **Triglycerides, LDL-1** | 5.92 | 4.25 | *** | *** |
| **Triglycerides, LDL-2** | 2.78 | 2.34 | *** | *** |
| **Triglycerides, LDL-3** | 2.65 | 1.95 | *** | *** |
| **Triglycerides, LDL-4** | 3.27 | 2.25 | *** | *** |
| **Triglycerides, LDL-5** | 3.3 | 2.27 | *** | *** |
| **Triglycerides, LDL-6** | 3.94 | 3.6 | * | * |
| **Cholesterol, LDL-1** | 17.13 | 12.17 | *** | *** |
| **Cholesterol, LDL-2** | 14.6 | 11.62 | *** | *** |
| **Cholesterol, LDL-3** | 14.57 | 9.97 | *** | *** |
| **Cholesterol, LDL-4** | 16.88 | 9.71 | *** | *** |
| **Cholesterol, LDL-5** | 20.11 | 13.33 | *** | *** |
| **Cholesterol, LDL-6** | 21.2 | 16.67 | *** | *** |
| **Free Cholesterol, LDL-1** | 5.94 | 4.54 | *** | *** |
| **Free Cholesterol, LDL-2** | 5.48 | 4.7 | *** | *** |
| **Free Cholesterol, LDL-3** | 5.4 | 4.53 | *** | *** |
| **Free Cholesterol, LDL-4** | 5.4 | 3.82 | *** | *** |
| **Free Cholesterol, LDL-5** | 5.69 | 4.17 | *** | *** |
| **Free Cholesterol, LDL-6** | 5.53 | 4.78 | *** | *** |
| **Phospholipids, LDL-1** | 10.46 | 8.48 | *** | *** |
| **Phospholipids, LDL-2** | 8.54 | 6.71 | *** | *** |
| **Phospholipids, LDL-3** | 8.05 | 6.35 | *** | *** |
| **Phospholipids, LDL-4** | 9.45 | 5.74 | *** | *** |
| **Phospholipids, LDL-5** | 10.99 | 7.71 | *** | *** |
| **Phospholipids, LDL-6** | 11.91 | 10.1 | *** | *** |
| **Apo-B, LDL-1** | 10.03 | 7.85 | *** | *** |
| **Apo-B, LDL-2** | 8.42 | 6.75 | *** | *** |
| **Apo-B, LDL-3** | 9.12 | 6.75 | *** | *** |
| **Apo-B, LDL-4** | 11.47 | 7.38 | *** | *** |
| **Apo-B, LDL-5** | 14.29 | 9.92 | *** | *** |
| **Apo-B, LDL-6** | 17.15 | 13.32 | *** | *** |
| **Triglycerides, HDL-1** | 3.92 | 4.38 | ns | ns |
| **Triglycerides, HDL-2** | 2.09 | 2.11 | ns | ns |
| **Triglycerides, HDL-3** | 2.46 | 2.39 | ns | ns |
| **Triglycerides, HDL-4** | 4.08 | 4.02 | ns | ns |
| **Cholesterol, HDL-1** | 15.99 | 17.47 | ns | ns |
| **Cholesterol, HDL-2** | 7.4 | 7.68 | ns | ns |
| **Cholesterol, HDL-3** | 8.82 | 9.16 | ns | ns |
| **Cholesterol, HDL-4** | 19.71 | 19.18 | ns | ns |
| **Free Cholesterol, HDL-1** | 4.78 | 4.91 | ns | ns |
| **Free Cholesterol, HDL-2** | 2.01 | 1.99 | ns | ns |
| **Free Cholesterol, HDL-3** | 2.16 | 2.25 | * | * |
| **Free Cholesterol, HDL-4** | 3.77 | 3.65 | ns | ns |
| **Phospholipids, HDL-1** | 16.74 | 20.55 | ** | * |
| **Phospholipids, HDL-2** | 11.04 | 11.65 | ns | ns |
| **Phospholipids, HDL-3** | 13.29 | 13.77 | ns | ns |
| **Phospholipids, HDL-4** | 24.72 | 25.47 | ns | ns |
| **Apo-A1, HDL-1** | 20.26 | 25.89 | ** | ** |
| **Apo-A1, HDL-2** | 17.17 | 18.96 | ** | ** |
| **Apo-A1, HDL-3** | 23.89 | 24.29 | ns | ns |
| **Apo-A1, HDL-4** | 79.31 | 77.75 | ns | ns |
| **Apo-A2, HDL-1** | 2.36 | 2.6 | ns | ns |
| **Apo-A2, HDL-2** | 3.42 | 3.45 | ns | ns |
| **Apo-A2, HDL-3** | 6.29 | 6.04 | ns | ns |
| **Apo-A2, HDL-4** | 20.5 | 19.36 | ns | ns |

**Supplementary Table 2.** Univariate analysis of quantified lipoproteins in each KODAMA group. Trimethylamine-N-oxide is reported in this table as the only significant metabolite. Median values of particle number parameters are expressed in nmol/L, Trimethylamine-N-oxide in mmol/L and all others lipoprotein parameters in mg/dL. The p-values obtained using Wilcoxon signed-rank test are provided also adjusted for FDR are reported. *** p < 0.001; ** p < 0.01; * p < 0.05; ns p ≥ 0.05.

|  | ***G1*** | | | ***G2*** | | | ***G3*** | | |
| --- | --- | --- | --- | --- | --- | --- | --- | --- | --- |
|  | **Median t0** | **Median t1** | **FDR adj. *P*-value** | **Median t0** | **Median t1** | **FDR adj. *P*-value** | **Median t0** | **Median t1** | **FDR adj. *P*-value** |
| **Trimethylamine-N-oxide** | 0.032 | 0.044 | ns | 0.038 | 0.038 | ns | 0.013 | 0.03 | * |
| **Main Parameter, TG** | 118.615 | 95.52 | * | 86.88 | 116.65 | ns | 159.925 | 148.405 | ** |
| **Main Parameters, Chol** | 167.435 | 130.615 | *** | 167.09 | 168.06 | ns | 285.135 | 172.985 | *** |
| **Main Parameters, LDL-Chol** | 92.48 | 53.02 | *** | 90.53 | 88.96 | ns | 160.695 | 87.8 | *** |
| **Main Parameters, HDL-Chol** | 47.125 | 49.98 | ns | 56.27 | 56.9 | ns | 55.405 | 55.58 | ns |
| **Main Parameters, Apo-A1** | 134.835 | 138.345 | ns | 158.37 | 151.26 | ns | 152.245 | 161.57 | ns |
| **Main Parameters, Apo-A2** | 31.41 | 31.705 | ns | 34.08 | 31.12 | ns | 34.77 | 35.57 | ns |
| **Main Parameters, Apo-B100** | 78.27 | 52.035 | *** | 71.33 | 73.79 | ns | 131.065 | 78.2 | *** |
| **LDL Chol to HDL Chol ratio** | 1.935 | 1.145 | *** | 1.64 | 1.61 | ns | 3.275 | 1.475 | *** |
| **Apo-B100 to Apo-A1 ratio** | 0.57 | 0.385 | *** | 0.48 | 0.49 | ns | 0.895 | 0.455 | *** |
| **Total Particle Number** | 1423.205 | 946.09 | *** | 1297.05 | 1341.62 | ns | 2383.11 | 1421.815 | *** |
| **VLDL Particle Number** | 168.23 | 129.06 | ** | 116.12 | 146.58 | ns | 199.045 | 174.115 | ** |
| **IDL Particle Number** | 107.085 | 80.595 | ** | 82.27 | 87.11 | ns | 182.77 | 101.615 | *** |
| **LDL Particle Number** | 1093.72 | 626 | *** | 1041.81 | 1037.7 | ns | 1940.285 | 1134.275 | *** |
| **LDL-1 Particle Number** | 147.82 | 135.445 | ns | 158.47 | 144.02 | ns | 286.1 | 145.785 | *** |
| **LDL-2 Particle Number** | 146.065 | 111.99 | ns | 148.42 | 130.66 | ns | 203.615 | 103.23 | *** |
| **LDL-3 Particle Number** | 138.53 | 107.005 | ns | 160.35 | 149.21 | ns | 266.025 | 136.465 | *** |
| **LDL-4 Particle Number** | 171.54 | 84.205 | ** | 164.13 | 125.15 | ns | 337.17 | 145.915 | *** |
| **LDL-5 Particle Number** | 253.09 | 128.15 | *** | 216.21 | 194.72 | ns | 321.785 | 228.43 | ** |
| **LDL-6 Particle Number** | 326.525 | 212.605 | *** | 258.13 | 265.26 | ns | 336.86 | 288.1 | ns |
| **Triglycerides, VLDL** | 85.855 | 58.015 | ** | 53.19 | 78.1 | ns | 100.965 | 104.665 | ns |
| **Triglycerides, IDL** | 8.175 | 5.225 | ** | 5.04 | 7.86 | ns | 14.275 | 13.045 | * |
| **Triglycerides, LDL** | 21.705 | 16.72 | *** | 21.27 | 20.55 | ns | 30.105 | 21.04 | *** |
| **Triglycerides, HDL** | 11.4 | 11.525 | ns | 11.33 | 12.23 | ns | 13.97 | 13.22 | ns |
| **Cholesterol, VLDL** | 21.055 | 12.955 | ns | 12.05 | 16.56 | ns | 28.275 | 21.145 | ** |
| **Cholesterol, IDL** | 11.55 | 8.96 | ** | 9.96 | 10.51 | ns | 25.225 | 11.095 | *** |
| **Free Cholesterol, VLDL** | 9.32 | 6.43 | * | 5.84 | 8.53 | ns | 11.705 | 10.415 | ** |
| **Free Cholesterol, IDL** | 3.315 | 2.37 | ** | 2.58 | 2.73 | ns | 7.025 | 3.1 | *** |
| **Free Cholesterol, LDL** | 29.845 | 19.96 | *** | 30.82 | 29.25 | ns | 47.965 | 29.035 | *** |
| **Free Cholesterol, HDL** | 11.625 | 11.93 | ns | 14.93 | 15.78 | ns | 14.24 | 14.51 | ns |
| **Phospholipids, VLDL** | 21.39 | 15.095 | * | 13.36 | 17.66 | ns | 24.81 | 24.78 | * |
| **Phospholipids, IDL** | 6.365 | 3.64 | *** | 5.55 | 6.04 | ns | 11.665 | 5.825 | *** |
| **Phospholipids, LDL** | 52.305 | 33.92 | *** | 53.45 | 49.47 | ns | 90.03 | 50.955 | *** |
| **Phospholipids, HDL** | 59.895 | 67.085 | ns | 72.55 | 73.11 | ns | 67.44 | 73.02 | ns |
| **Apo-A1, HDL** | 130.17 | 135.165 | ns | 151.82 | 148.79 | ns | 148.385 | 158.385 | ns |
| **Apo-A2, HDL** | 32.055 | 31.985 | ns | 34.61 | 32.28 | ns | 34.97 | 35.3 | ns |
| **Apo-B, VLDL** | 9.25 | 7.095 | ** | 6.39 | 8.06 | ns | 10.945 | 9.58 | ** |
| **Apo-B, IDL** | 5.89 | 4.43 | ** | 4.52 | 4.79 | ns | 10.05 | 5.585 | *** |
| **Apo-B, LDL** | 60.155 | 34.43 | *** | 57.3 | 57.07 | ns | 106.71 | 62.38 | *** |
| **Triglycerides, VLDL-1** | 43.08 | 30.705 | ns | 25.18 | 43.04 | ns | 47.89 | 53.045 | ns |
| **Triglycerides, VLDL-2** | 13.3 | 7.07 | ** | 7.08 | 9.4 | ns | 15.095 | 11.655 | ns |
| **Triglycerides, VLDL-3** | 13.335 | 7.475 | ** | 8.16 | 8.99 | ns | 12.98 | 10.76 | ns |
| **Triglycerides, VLDL-4** | 10.085 | 8.14 | ** | 7.19 | 8.36 | ns | 11.52 | 8.68 | ** |
| **Triglycerides, VLDL-5** | 2.32 | 2.255 | ns | 2.29 | 2.37 | ns | 2.99 | 2.575 | ** |
| **Cholesterol, VLDL-1** | 5.53 | 4.135 | ns | 3.2 | 5.05 | ns | 8.715 | 7.1 | * |
| **Cholesterol, VLDL-2** | 2.99 | 1.18 | ** | 1.66 | 2.05 | ns | 3.595 | 2.065 | * |
| **Cholesterol, VLDL-3** | 3.9 | 2.35 | ** | 2.24 | 2.67 | ns | 4.295 | 3.02 | * |
| **Cholesterol, VLDL-4** | 4.775 | 3.525 | ns | 3.39 | 3.17 | ns | 7.05 | 3.76 | *** |
| **Cholesterol, VLDL-5** | 0.595 | 0.41 | ns | 0.46 | 0.67 | ns | 1.095 | 0.605 | ** |
| **Free Cholesterol, VLDL-1** | 1.99 | 1.125 | ns | 0.85 | 1.49 | ns | 2.9 | 2.74 | ns |
| **Free Cholesterol, VLDL-2** | 1.535 | 0.98 | ** | 0.65 | 1.35 | ns | 1.72 | 1.495 | * |
| **Free Cholesterol, VLDL-3** | 1.9 | 1.05 | * | 0.8 | 1.39 | ns | 2.065 | 1.82 | ** |
| **Free Cholesterol, VLDL-4** | 2.025 | 1.62 | ns | 1.59 | 1.52 | ns | 3.575 | 1.92 | *** |
| **Free Cholesterol, VLDL-5** | 0.06 | 0.17 | ns | 0.14 | 0.23 | ns | 0.78 | 0.39 | ** |
| **Phospholipids, VLDL-1** | 6.365 | 5.035 | ns | 3.83 | 6.27 | ns | 7.725 | 7.805 | ns |
| **Phospholipids, VLDL-2** | 3.53 | 2.005 | *** | 1.91 | 2.37 | ns | 3.96 | 2.86 | ns |
| **Phospholipids, VLDL-3** | 4.575 | 3.015 | ** | 2.57 | 3.19 | ns | 4.23 | 3.435 | ns |
| **Phospholipids, VLDL-4** | 4.805 | 3.805 | * | 3.69 | 3.7 | ns | 6.095 | 4.12 | *** |
| **Phospholipids, VLDL-5** | 1.065 | 0.785 | ns | 0.88 | 0.92 | ns | 1.495 | 1.22 | * |
| **Triglycerides, LDL-1** | 4.68 | 3.17 | *** | 4.39 | 4.74 | ns | 7.96 | 4.92 | *** |
| **Triglycerides, LDL-2** | 2.525 | 2.38 | ns | 2.46 | 2.48 | ns | 3.84 | 2.21 | *** |
| **Triglycerides, LDL-3** | 2.29 | 1.58 | ** | 2.15 | 2 | ns | 3.46 | 2.02 | *** |
| **Triglycerides, LDL-4** | 2.83 | 1.615 | ** | 2.63 | 2.31 | ns | 4.07 | 2.44 | *** |
| **Triglycerides, LDL-5** | 3.025 | 1.585 | *** | 2.61 | 2.53 | ns | 3.835 | 2.515 | ** |
| **Triglycerides, LDL-6** | 3.95 | 2.78 | *** | 3.69 | 4.02 | ns | 4.16 | 4.495 | ns |
| **Cholesterol, LDL-1** | 12.64 | 11.97 | ns | 15.22 | 11.84 | ns | 29.03 | 13.11 | *** |
| **Cholesterol, LDL-2** | 13.305 | 10.105 | ns | 14.6 | 11.78 | ns | 17.18 | 7.99 | *** |
| **Cholesterol, LDL-3** | 11.765 | 8.07 | ns | 13.2 | 11.15 | ns | 22.765 | 10.645 | *** |
| **Cholesterol, LDL-4** | 13.295 | 6.02 | ** | 12.92 | 9.02 | ns | 27.575 | 12.215 | *** |
| **Cholesterol, LDL-5** | 19.565 | 9.775 | *** | 14.13 | 14.71 | ns | 24.63 | 17.18 | ** |
| **Cholesterol, LDL-6** | 21.44 | 14.34 | *** | 17.34 | 18.46 | ns | 23.855 | 19.475 | ns |
| **Free Cholesterol, LDL-1** | 4.42 | 4.205 | ns | 5.15 | 4.6 | ns | 9.045 | 5.235 | *** |
| **Free Cholesterol, LDL-2** | 5.185 | 4.435 | ns | 5.55 | 5.31 | ns | 6.23 | 3.915 | *** |
| **Free Cholesterol, LDL-3** | 4.485 | 3.615 | ns | 5.46 | 5.11 | ns | 7.13 | 4.44 | *** |
| **Free Cholesterol, LDL-4** | 4.46 | 2.875 | ** | 4.87 | 4.07 | ns | 8.54 | 4.885 | *** |
| **Free Cholesterol, LDL-5** | 5.375 | 3.08 | *** | 5.08 | 4.48 | ns | 7.5 | 4.95 | *** |
| **Free Cholesterol, LDL-6** | 5.685 | 4.3 | *** | 5.1 | 5.49 | ns | 6.2 | 5.285 | ns |
| **Phospholipids, LDL-1** | 8.52 | 8.46 | ns | 9.64 | 8.48 | ns | 16.2 | 8.685 | *** |
| **Phospholipids, LDL-2** | 7.63 | 6.21 | ns | 8.54 | 7.2 | ns | 10.225 | 5.6 | *** |
| **Phospholipids, LDL-3** | 6.965 | 5.46 | ns | 7.93 | 7.12 | ns | 12.98 | 7.03 | *** |
| **Phospholipids, LDL-4** | 7.535 | 3.75 | ** | 7.53 | 5.49 | ns | 14.915 | 7.07 | *** |
| **Phospholipids, LDL-5** | 10.745 | 5.81 | *** | 8.41 | 8.18 | ns | 13.405 | 9.73 | ** |
| **Phospholipids, LDL-6** | 12.195 | 8.485 | *** | 10.51 | 10.85 | ns | 13.375 | 11.305 | ns |
| **Apo-B, LDL-1** | 8.13 | 7.45 | ns | 8.72 | 7.92 | ns | 15.735 | 8.02 | *** |
| **Apo-B, LDL-2** | 8.035 | 6.16 | ns | 8.16 | 7.19 | ns | 11.2 | 5.675 | ** |
| **Apo-B, LDL-3** | 7.615 | 5.885 | ns | 8.82 | 8.21 | ns | 14.63 | 7.505 | *** |
| **Apo-B, LDL-4** | 9.435 | 4.63 | ** | 9.03 | 6.88 | ns | 18.54 | 8.025 | *** |
| **Apo-B, LDL-5** | 13.915 | 7.045 | *** | 11.89 | 10.71 | ns | 17.695 | 12.565 | ** |
| **Apo-B, LDL-6** | 17.96 | 11.695 | *** | 14.2 | 14.59 | ns | 18.53 | 15.845 | ns |
| **Triglycerides, HDL-1** | 3.51 | 3.61 | ns | 3.92 | 4.7 | ns | 4.47 | 4.505 | ns |
| **Triglycerides, HDL-2** | 1.83 | 1.915 | ns | 1.95 | 2.08 | ns | 2.325 | 2.35 | ns |
| **Triglycerides, HDL-3** | 2.285 | 2.315 | ns | 2.1 | 2.17 | ns | 2.78 | 2.665 | ns |
| **Triglycerides, HDL-4** | 4.08 | 3.6 | ns | 3.56 | 3.71 | ns | 4.685 | 4.45 | ns |
| **Cholesterol, HDL-1** | 12.97 | 15.305 | ns | 19.42 | 21.8 | ns | 15.615 | 17.29 | ns |
| **Cholesterol, HDL-2** | 6.685 | 7.105 | ns | 8.03 | 7.82 | ns | 7.07 | 7.52 | ns |
| **Cholesterol, HDL-3** | 7.99 | 8.35 | ns | 9.38 | 9.44 | ns | 8.905 | 9.965 | ns |
| **Cholesterol, HDL-4** | 19.83 | 18.82 | ns | 18.94 | 17.72 | ns | 19.895 | 21.215 | ns |
| **Free Cholesterol, HDL-1** | 4.14 | 3.785 | ns | 5.84 | 6.01 | ns | 4.78 | 4.845 | ns |
| **Free Cholesterol, HDL-2** | 1.705 | 1.905 | ns | 2.26 | 2.07 | ns | 2.04 | 2.035 | ns |
| **Free Cholesterol, HDL-3** | 1.95 | 2.005 | ns | 2.2 | 2.17 | ns | 2.645 | 2.445 | ns |
| **Free Cholesterol, HDL-4** | 3.46 | 3.4 | ns | 3.77 | 3.65 | ns | 4.175 | 4.305 | ns |
| **Phospholipids, HDL-1** | 13.945 | 17.09 | ns | 22.84 | 23.86 | ns | 15.31 | 19.885 | ns |
| **Phospholipids, HDL-2** | 9.965 | 10.785 | ns | 12.3 | 12.74 | ns | 10.43 | 12.345 | ns |
| **Phospholipids, HDL-3** | 11.9 | 13.09 | ns | 14.16 | 14.32 | ns | 13.525 | 15.535 | ns |
| **Phospholipids, HDL-4** | 23.955 | 23.595 | ns | 24.47 | 22.48 | ns | 27.425 | 27.815 | ns |
| **Apo-A1, HDL-1** | 17.305 | 21.85 | ns | 29.8 | 33.13 | ns | 17.54 | 25.055 | * |
| **Apo-A1, HDL-2** | 15.605 | 17.34 | ns | 20.78 | 20.88 | ns | 16.655 | 20.155 | ** |
| **Apo-A1, HDL-3** | 20.76 | 23.395 | ns | 25.02 | 24.43 | ns | 25.04 | 26.995 | ns |
| **Apo-A1, HDL-4** | 76.855 | 75.455 | ns | 75.31 | 72.77 | ns | 83.655 | 84.05 | ns |
| **Apo-A2, HDL-1** | 1.82 | 1.78 | ns | 3.08 | 2.95 | ns | 2.2 | 2.335 | ns |
| **Apo-A2, HDL-2** | 3.06 | 3.23 | ns | 3.6 | 3.6 | ns | 3.425 | 3.455 | ns |
| **Apo-A2, HDL-3** | 5.59 | 5.81 | ns | 6.29 | 5.95 | ns | 6.75 | 6.21 | ns |
| **Apo-A2, HDL-4** | 20.82 | 19.3 | ns | 18.94 | 17.92 | ns | 21.045 | 20.66 | ns |


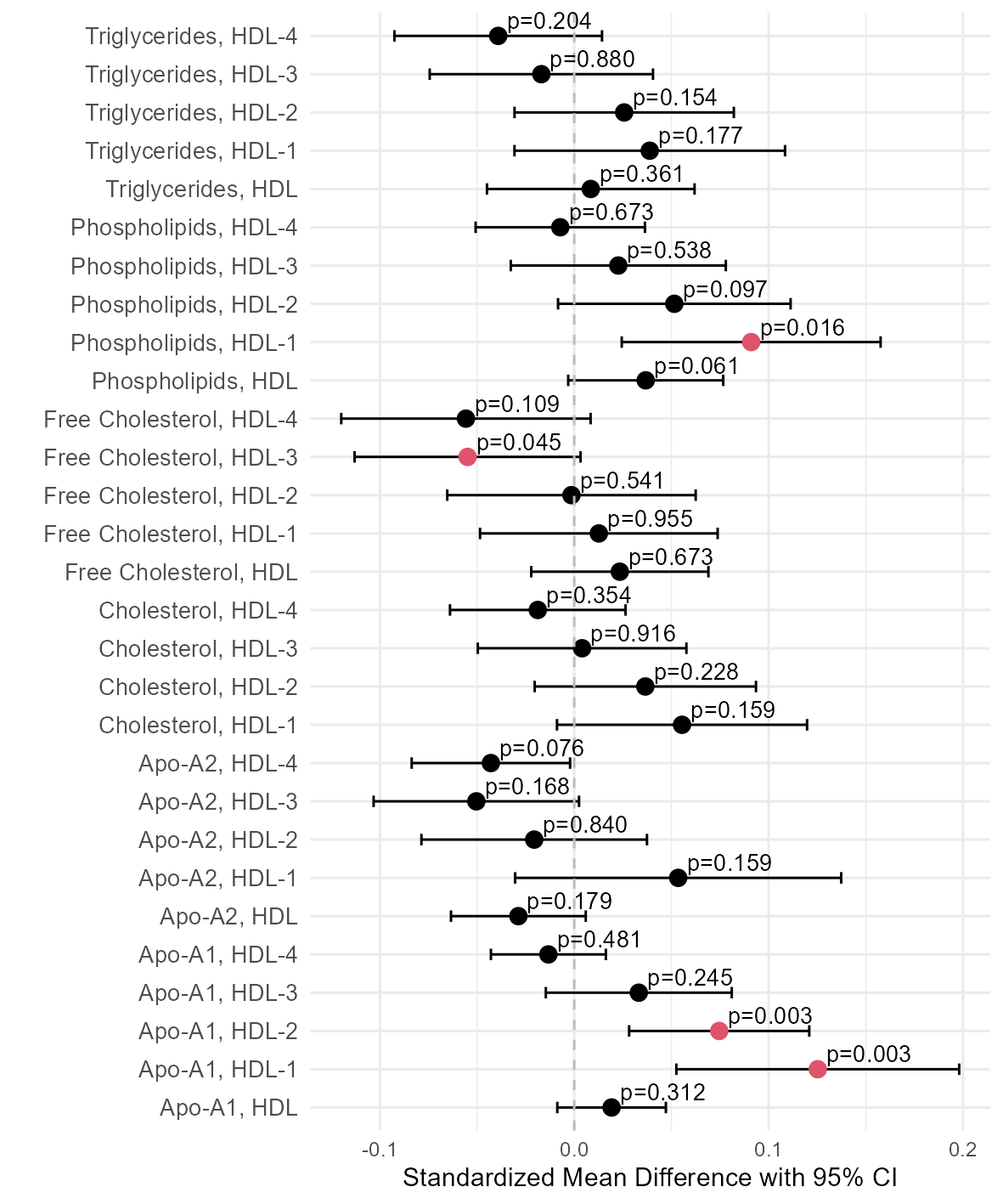
**Supplementary Figure 1.** HDL-related parameters standardized mean differences between t1 and t0 samples. A negative value means decreasing at t1. Red dots denote the significant (p FDR adjusted < 0.05) parameters.


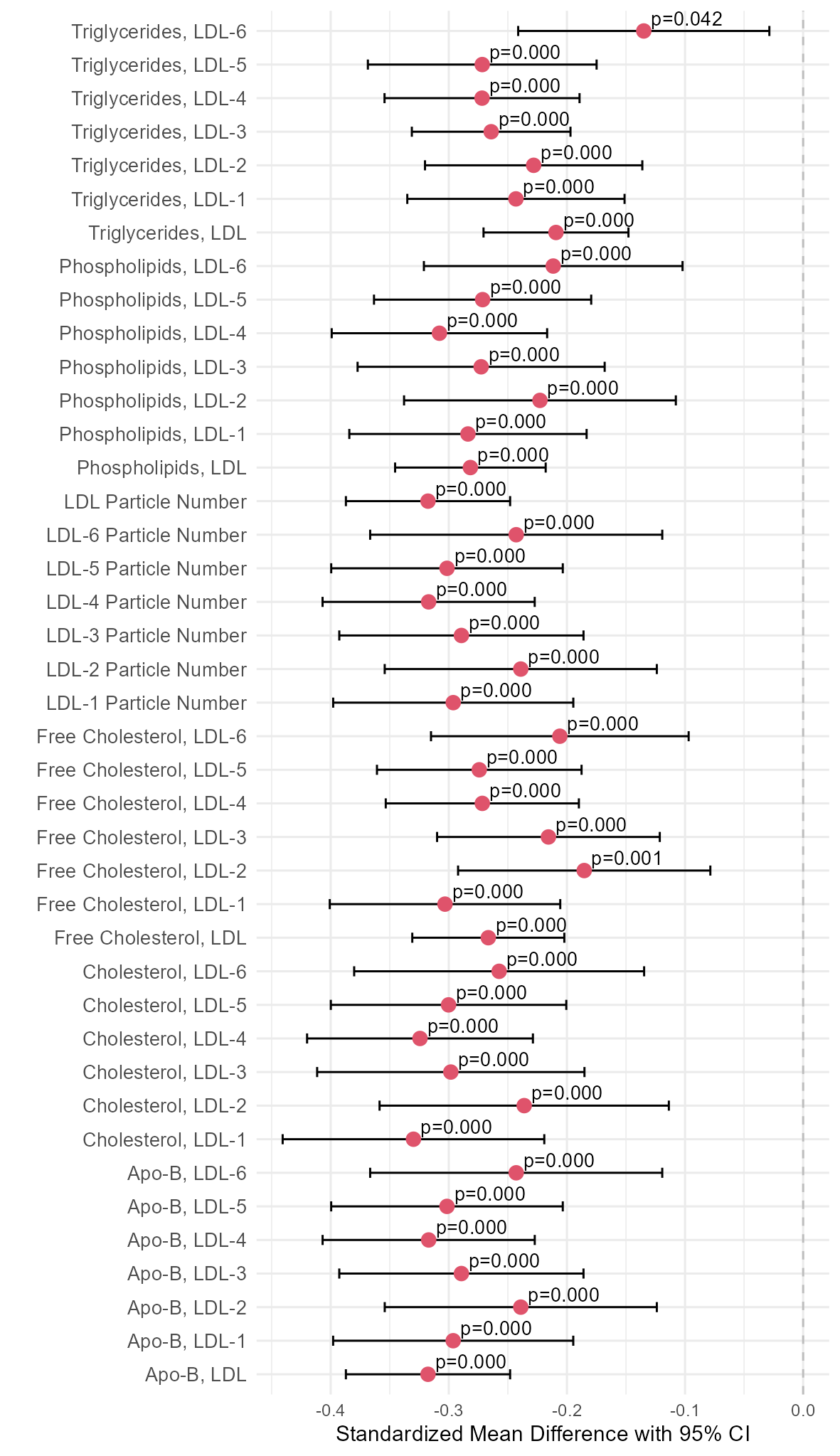
**Supplementary Figure 2.** LDL-related parameters standardized mean differences between t1 and t0 samples. A negative value means decreasing at t1. Red dots denote the significant (p FDR adjusted < 0.05) parameters.


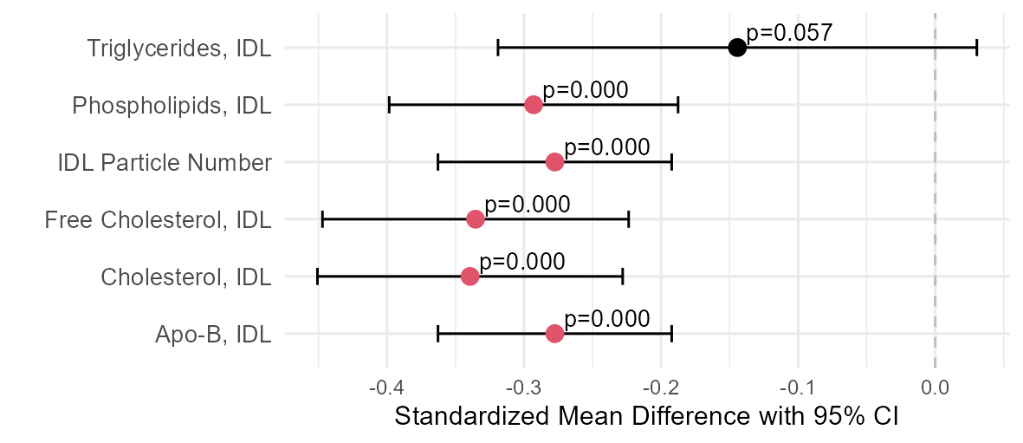
**Supplementary Figure 3.** IDL-related parameters standardized mean differences between t1 and t0 samples. A negative value means decreasing at t1. Red dots denote the significant (p FDR adjusted < 0.05) parameters.


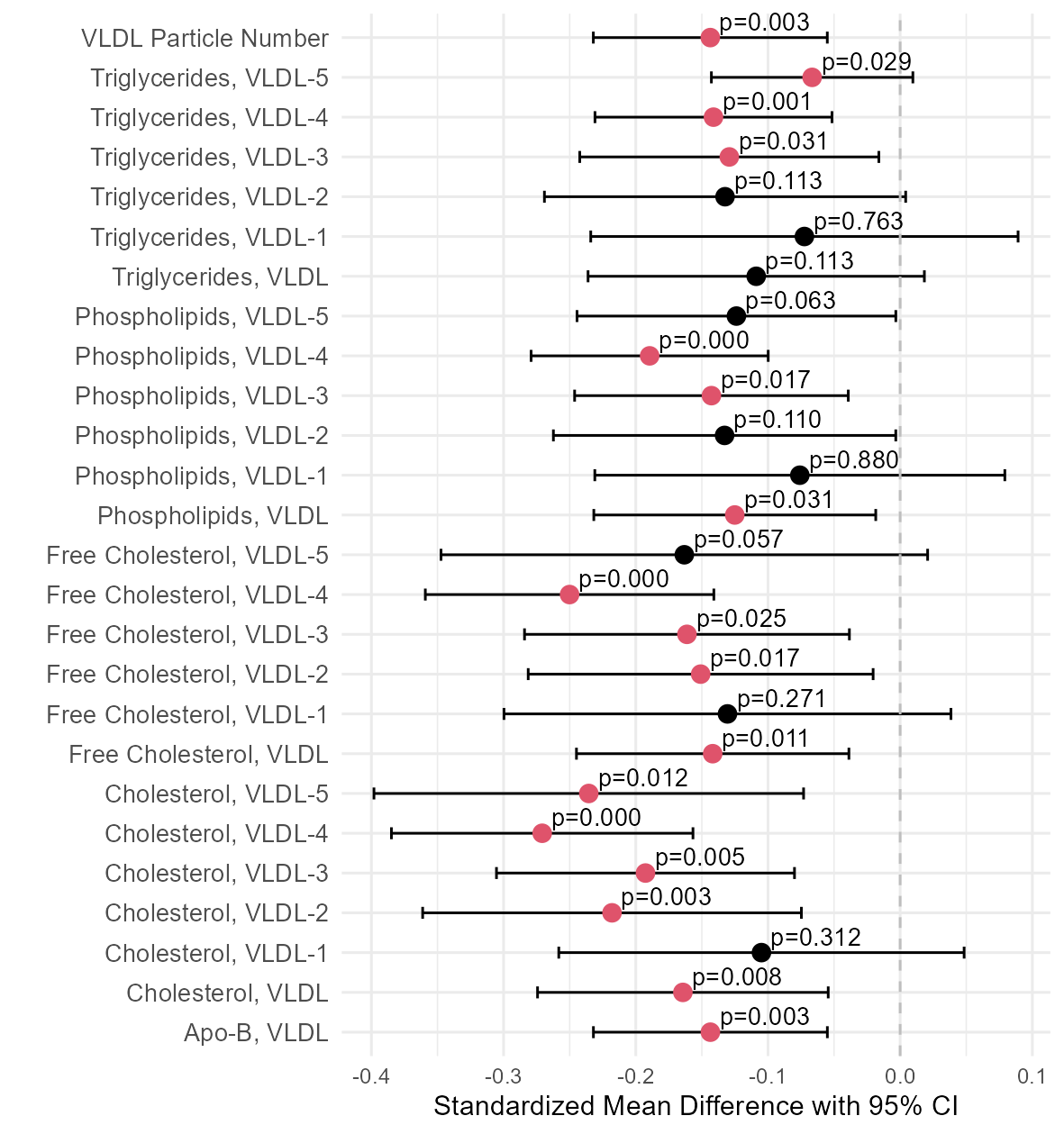


**Supplementary Figure 4.** VLDL-related parameters standardized mean differences between t1 and t0 samples. A negative value means decreasing at t1. Red dots denote the significant (p FDR adjusted < 0.05) parameters.
